# Enzyme-Assisted Extraction of Polysaccharides from Steam-Exploded *Ganoderma lucidum* and Its Yield, Structural Characterisation, and Immunomodulatory Activity

**DOI:** 10.3390/molecules31111864

**Published:** 2026-05-29

**Authors:** Jing Liu, Zhihao Yang, Jiamin Huang, Chong Sun, Lijing Chen, Zhenyuan Zhu

**Affiliations:** 1 State Key Laboratory of Food Nutrition and Safety, Tianjin University of Science and Technology, Tianjin 300457, China; 18702280701@163.com (J.L.); 13821801408@163.com (Z.Y.); 16629068382@163.com (J.H.); 15613402005@163.com (C.S.); 2 Key Laboratory of Food Nutrition and Safety, Ministry of Education, Tianjin University of Science and Technology, Tianjin 300457, China; 3 College of Food Science and Engineering, Tianjin University of Science and Technology, Tianjin 300457, China; 4 Department of Biomedicine and Health Sciences, Shanghai Vocational College of Agriculture and Forestry, Shanghai 201699, China; 212215@shafc.edu.cn

**Keywords:** *Ganoderma lucidum*, enzymatic extraction, polysaccharide, structural characteristic, immunomodulatory activity

## Abstract

Polysaccharides are the primary bioactive constituents of *Ganoderma lucidum*. Nevertheless, the industrial potential of this species is constrained by the fact that yields are relatively low. The present study developed a technology and method of extracting polysaccharides from steam-exploded *Ganoderma lucidum* by enzymatic extraction, with the aim of increasing the yield. The yield of crude polysaccharide is 15.39%, which is 6.96 times that of the traditional water method (2.21%). A purified, homogeneous polysaccharide fraction, designated GLP-L, was isolated with a molecular weight of 5.59 × 10^6^ Da. A detailed analysis of the chemical structure of the substance was conducted, and its immunomodulatory activity was thoroughly researched. A thorough investigation into the structural characteristics of GLP-L was conducted, yielding the following findings: GLP-L is composed of D-mannose (52.76 mol%), D-glucose (36.98 mol%), D-xylose (6.39 mol%), and D-galactose (3.87 mol%). The structure was characterised by methylation, nuclear magnetic resonance spectroscopy, and Fourier transform infrared spectroscopy. In the second step of the process, the following sequence of reactions occurs: 2)-β-D-Gal-(1→4)-α-D-Xyl*p*-(1→3,4)-α-D-Man*p*-(1→6)-β-D-Glc*p*-(1→4)-β-D-Man*p*-(1→4)-α-D-Glc*p*-(1→. The effect of GLP-L on immunomodulatory activity was evaluated through the RAW264.7 macrophage model, with the results showing an enhancement in phagocytic activity, immune-related enzyme activities, nitric oxide production, and cytokine secretion. The enzyme-assisted extraction method was demonstrated to enhance the yield of *Ganoderma lucidum* polysaccharides, with the extract displaying noteworthy immunomodulatory activity. This provides a novel strategy for the production of polysaccharide immunomodulators.

## 1. Introduction

*Ganoderma lucidum* is a well-known large medicinal and edible fungus with a long history [1,2]. The fruiting bodies of this species have been the subject of extensive research. It contains a rich diversity of components, including polysaccharides, triterpenoids, peptides, sterols, flavonoids, fatty acids, vitamins, and minerals [3,4,5]. *Ganoderma lucidum* polysaccharides (GLPs) are a class of water-soluble heteropolysaccharides, comprising monosaccharides such as glucose, mannose and galactose, with complex glycosidic bond connections and distinctive molecular structures. As demonstrated by experimental means, GLP exerts a variety of powerful biological activities. As demonstrated in the extant literature, the substance under discussion exerts a role in immune regulation, antioxidation, anti-inflammation and anti-tumour activity [6]. Immunomodulatory activity is a significant area of research focus for GLPs. As Wang et al. [7] reported, the immune microenvironment of a tumour can be reshaped by GLPs, with the result of T and B lymphocytes being activated and macrophages stimulated to a degree that renders the tumour cells more susceptible to destruction. As demonstrated by Gao et al. [8], GLP-1 has been shown to have immunomodulatory properties that are both effective and reliable.

Nevertheless, the yield of GLPs obtained by means of the conventional water extraction method is comparatively low. In order to optimise the utilisation of resources, it is imperative to enhance the efficiency of extraction methods without compromising immunomodulatory activity. This has emerged as a pivotal area of research. Wang et al. extracted polysaccharides from Enteromorpha prolifera by means of an enzymatic extraction process, thereby increasing the extraction rate from 6.43% by water extraction to 26.68%. The extract thus obtained demonstrated strong biological activity [9]. Chu et al. confirmed that the samples subjected to steam explosion pretreatment exhibited an augmented polysaccharide release rate [10]. Lin et al. extracted StPP by means of physical treatment enzyme-assisted extraction. The extraction rate (30.13%) was found to be significantly higher than that of the water extraction method [11]. As demonstrated in the relevant literature, the extraction of polysaccharides through the use of enzymes has been shown to be a highly effective method of extraction [12,13]. As demonstrated in [14], polysaccharides are closely associated with cellulose, hemicellulose, lignin and other matrix components of fungal cell walls. The dense cell wall structure forms a physical barrier to polysaccharide dissolution. The barrier hinders the diffusion of polysaccharides into the extraction solvent. The enzyme extraction method has been shown to target and degrade the key components that form the physical barrier of the cell wall by adding specific biological enzymes to the extraction system [15]. In the process of polysaccharide extraction, the combination of physical pretreatment and enzymatic hydrolysis has been shown to produce a synergistic effect [16]. The consequence of this process is the acceleration of contact between enzyme preparations and raw materials, an increase in the rate of enzymatic reactions, and a reduction in the time required for enzymatic hydrolysis. The crude fibre content of the fruiting body of *Ganoderma lucidum* is comparatively elevated. The process of steam explosion (SE) has been demonstrated to be capable of hydrolysing and softening both hemicellulose and lignin. The cell wall is destroyed by the mechanical force generated by the instantaneous explosion. Furthermore, SE has been demonstrated to be an efficient, cost-effective, and energy-saving method that can be applied in industrial production [17]. In summary, the enzyme-assisted extraction method has been demonstrated to increase the yield of GLPs and achieve a synergistic enhancement effect.

The present study aims to adopt a novel method combining steam explosion pretreatment and compound enzyme-assisted extraction to obtain GLPs. The impact of this method on polysaccharide extraction yield will be explored, and the structural composition of the resultant GLPs will be characterised in greater detail. The present study is founded upon the RAW 264.7 macrophage cell model. The immunomodulatory activity of the obtained GLPs is also evaluated in this work. The findings provide a theoretical basis for the efficient preparation, structural research and functional application of GLPs and offer a reference for the industrial production and deep utilisation of fungal polysaccharide resources.

## 2. Results and Discussion

The ensuing data set presents the results of the extraction yield of GLPs obtained by employing a variety of extraction methods. The extraction rate of GLP-CW is 2.21%, while that of GLP-CL is 15.39%. The extraction rate of crude polysaccharides via the enzyme-assisted extraction method is 6.96 times that of water extraction. It can thus be concluded that the enzyme-assisted extraction method is an efficient extraction method [18].

The molecular weight distribution of GLP-CL is illustrated in Figure S1. The chromatogram exhibited multiple peaks of varying areas, and the predominant main peak was selected for subsequent purification. The elution profile of DEAE-52 cellulose column purification is illustrated in Figure S2. The fraction eluted with distilled water demonstrated a substantial peak area. The obtained sample was then analysed using HPLC. The chromatogram (Figure S3) exhibited two peaks. The primary peak was subsequently subjected to a purification process, which involved the use of a Sephadex G-200 column. The elution profile is illustrated in Figure S4. The fractions exhibiting the highest absorption peaks were then pooled, collected, and subjected to freeze-drying, a process that resulted in the purification of a polysaccharide known as GLP-L.

**Figure 1:**
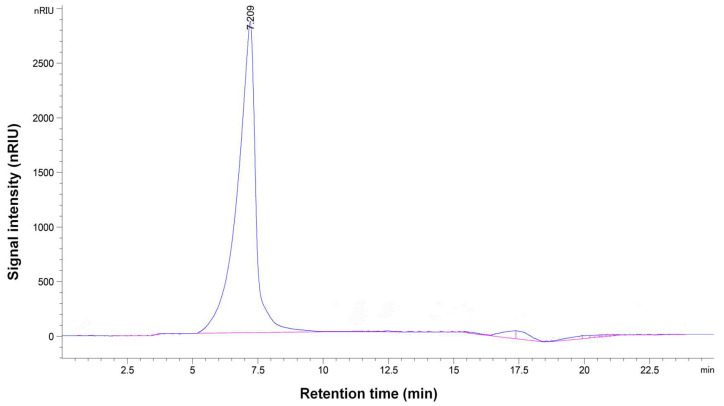
Molecular weight distribution of GLP-L.

As illustrated in Figure 1, a sharp and symmetrical single peak emerged at 7.209 min. Utilising the standard curve (y = −0.3374x + 9.177, where y = lgMw, x = retention time Rt, R^2^ = 0.9941) (Figure S5), the molecular weight of GLP-L is determined to be 5.59 × 10^6^ Da. However, UV-Vis spectrophotometry revealed no discernible characteristic absorption of GLP-L at 260 nm and 280 nm (Figure S6). The absence of nucleic acid and protein contamination was confirmed. The standard curve (y=10.641x − 0.009; R^2^ = 0.9991) (Figure S7) was utilised to determine the polysaccharide content, which was found to be 93.98 ± 0.02%. As demonstrated in Figure S8, which presents the galacturonic acid standard curve y = 10.731x − 0.0059 (R^2^ = 0.9992), the GLP-L sample exhibited minimal galacturonic acid content. The results of this study demonstrate that GLP-L is a high-molecular-weight, neutral, and homogeneous pure polysaccharide.

**Figure 2:**
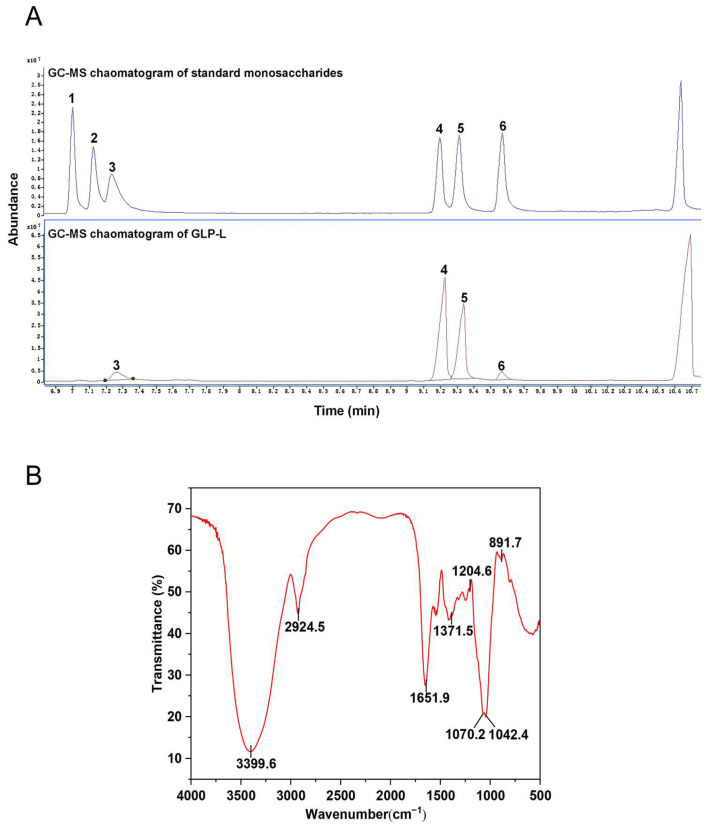
GC/MS chromatogram of monosaccharide composition of GLP-L. 1: Rha, 2: Ara, 3: Xyl, 4: Man, 5: Glc, 6: Gal. (**A**); FT-IR spectrum of GLP-L (**B**). GC/MS stands for gas chromatography–mass spectrometry; FT-IR stands for Fourier transform infrared spectroscopy.

The monosaccharide composition of GLP-L was determined via GC/MS analysis. The results are presented in Figure 2A. GLP-L was composed of D-mannose, D-glucose, D-xylose and D-galactose, with the molar percentages of 52.76%, 36.98%, 6.39%, and 3.87%, respectively. D-mannose was identified as the predominant component, and it was hypothesised that the conditions may facilitate the immunomodulatory activity of polysaccharides [19,20].

The chemical bond types, spatial configuration and characteristic functional groups of GLP-L were characterised by Fourier transform infrared (FT-IR) spectroscopy. The obtained spectral data are displayed in Figure 2B. The detailed spectral analysis results are as follows:

A broad absorption peak was observed at approximately 3440 cm^−1^, which is attributed to the stretching vibration of the O–H bond. An absorption peak was observed at approximately 2924 cm^−1^, which is attributed to the stretching vibration of the C–H bond. The peaks within the wavenumber range of 1720–1706 cm^−1^ are characteristic absorptions of acidic sugars, which were not detected. This result indicates that GLP-L is identified as a typical neutral polysaccharide. The absence of absorption bands at 1541 cm^−1^ in the sample confirmed that GLP-L is free from protein contamination and possesses high purity [21]. In the wavenumber range of 1400–1000 cm^−1^, characteristic vibration absorption was observed, attributed to glycosidic bonds (C–O–C) and hydroxyl groups (–OH). This finding is indicative of the presence of a pyranose ring structure [22]. Concurrently, the distinctive absorption peak at 890.969 cm^−1^ substantiates the presence of β-type glycosidic bonds within GLP-L.

GLP-L was subjected to methylation analysis. The data obtained from the methylation analysis of GLP-L was interpreted by comparing it with the standard spectra of some methylated sugar alcohol acetates in the database of the Complex Carbohydrate Research Center. The identification of glycosidic linkage types was achieved. GLP-L exhibits a diverse linkage profile (Table 1), comprising nine distinct linkages. The following elements were identified, and their structures were determined and are demonstrated in Figure S9: T-linked-Man*p*, 1,2-linked-Gal*f*, 1,4-linked-Glc*p*, 1,4-linked-Man*p*, 1,6-linked-Glc*p*, 1,4-linked-Xyl*p* and 1,3,4-linked-Man*p*. The molar percentages are found to be in close proximity to the proportions of the monosaccharide composition that were mentioned in the preceding paragraph.

**Figure 3:**
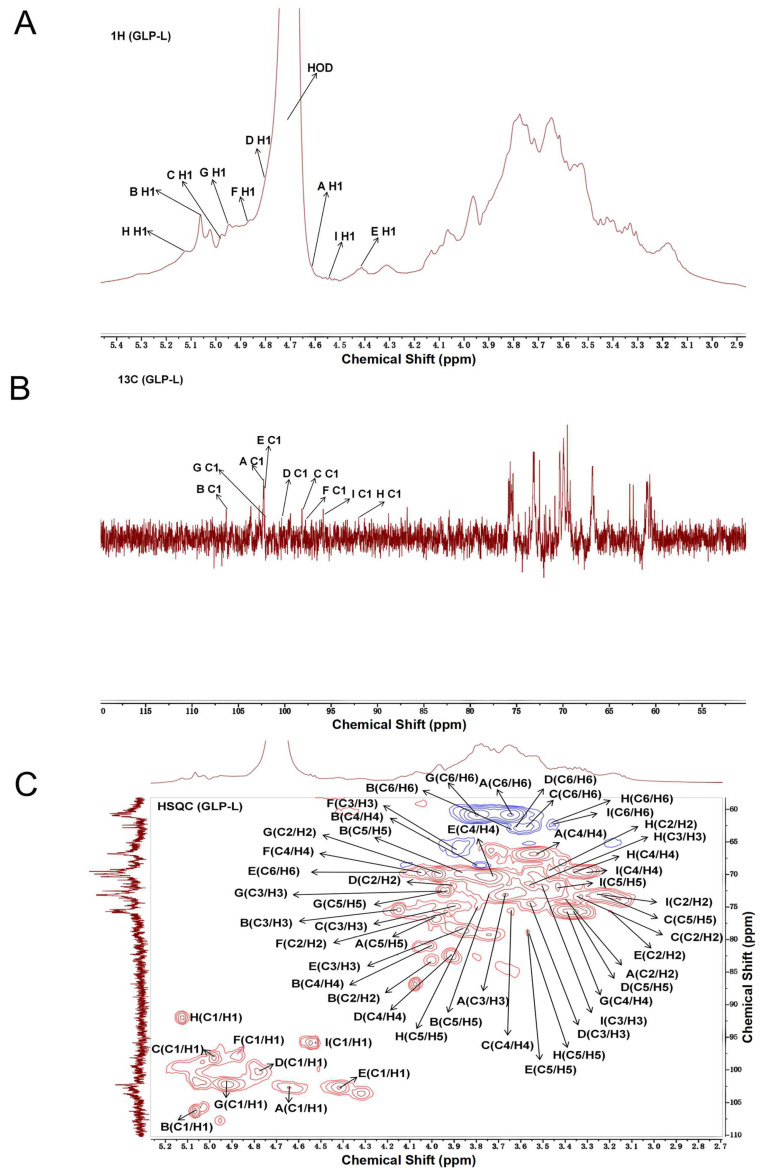
NMR spectra of GLP-L: 1H NMR (**A**); 13C NMR (**B**); HSQC (**C**). NMR stands for nuclear magnetic resonance.

As demonstrated in the hydrogen spectrum (3–5.9 ppm, Figure 3A) and the carbon spectrum (90–110 ppm, Figure 3B), GLP-L exhibits a complex structural architecture. In the HSQC spectrum of GLP-L (Figure 3C), nine correlation signals were identified at δH/δC 4.64/102.72, 5.06/106.27, 4.98/98.21, 4.78/100.30, 4.42/102.72, 4.87/97.89, 4.92/102.24, 5.12/92.08, and 4.54/95.79 ppm, which were assigned to glycosyl residues A–I: T-β-D-Man*p*, →2)-β-D-Gal*f*-(1→, →4)-α-D-Glc*p*-(1→, →4)-β-D-Man*p*-(1→, →6)-β-D-Glc*p*-(1→, →4)-α-D-Xyl*p*-(1→, →3,4)-α-D-Man*p*-(1→, α-reducing Glc*p*, and β-reducing Glc*p*. The structure of the compound under investigation contains the following elements: 6)-β-D-Glc*p*-(1→, →4)-α-D-Xyl*p*-(1→, →3,4)-α-D-Man*p*-(1→, α-reducing Glc*p*, and β-reducing Glc*p*. It is evident that these assignments are consistent with previously reported polysaccharide structures, as evidenced in the following references: [23,24,25,26,27,28,29,30,31]. The detailed chemical shifts for each residue are summarised in Table 2.

**Figure 4:**
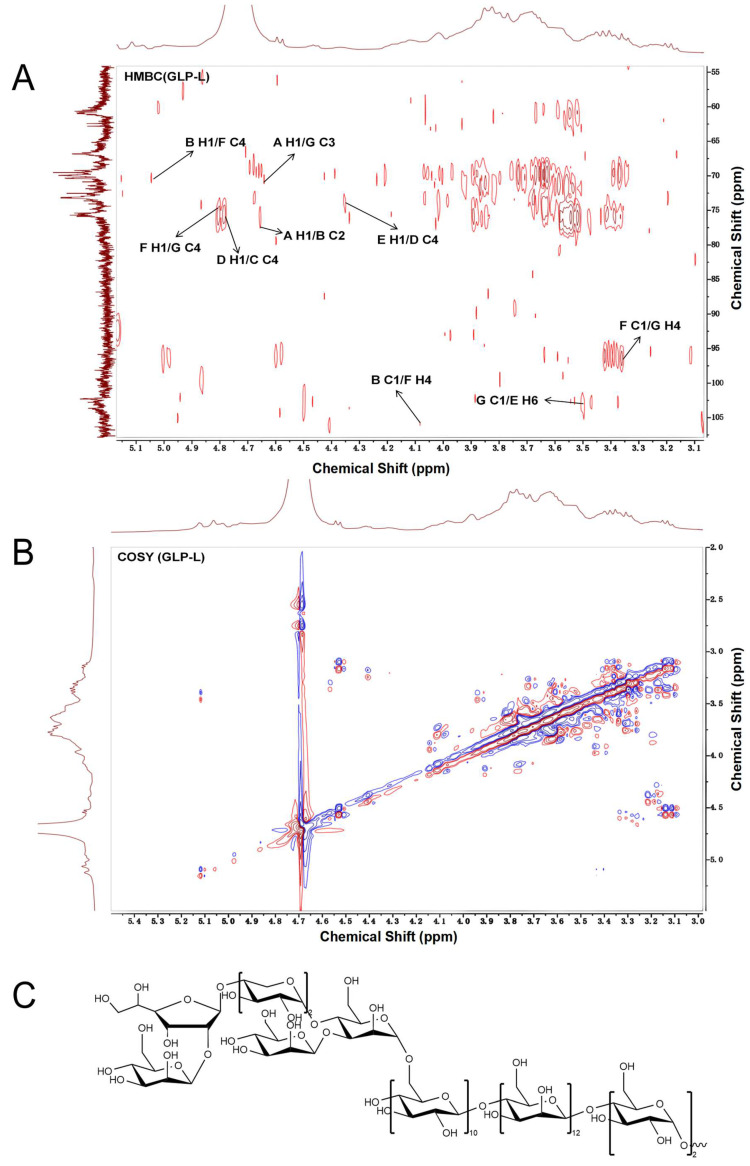
NMR spectra of GLP-L: HMBC (**A**); COSY (**B**); putative structure (**C**). NMR stands for nuclear magnetic resonance.

The application of HMBC spectroscopic analysis was undertaken for the purpose of investigating intra-residue correlations in GLP-L. In the second step of the process, the following sequence of reactions occurs, as demonstrated in Figure 4A: 2)-β-D-Gal-(1→4)-α-D-Xyl*p*-(1→3,4)-α-D-Man*p*-(1→6)-β-D-Glc*p*-(1→4)-β-D-Man*p*-(1→4)-α-D-Glc*p*-(1→. Cross-signals including AH1–GC3, AH1–BC2, BH1–FC4, FH1–GC4, DH1–CC4, EH1–DC4, BC1–FH4, GC1–EH6, and FC1–GH4 are also present. The proposed primary structure of GLP-L is illustrated in Figure 4C.

**Figure 5:**
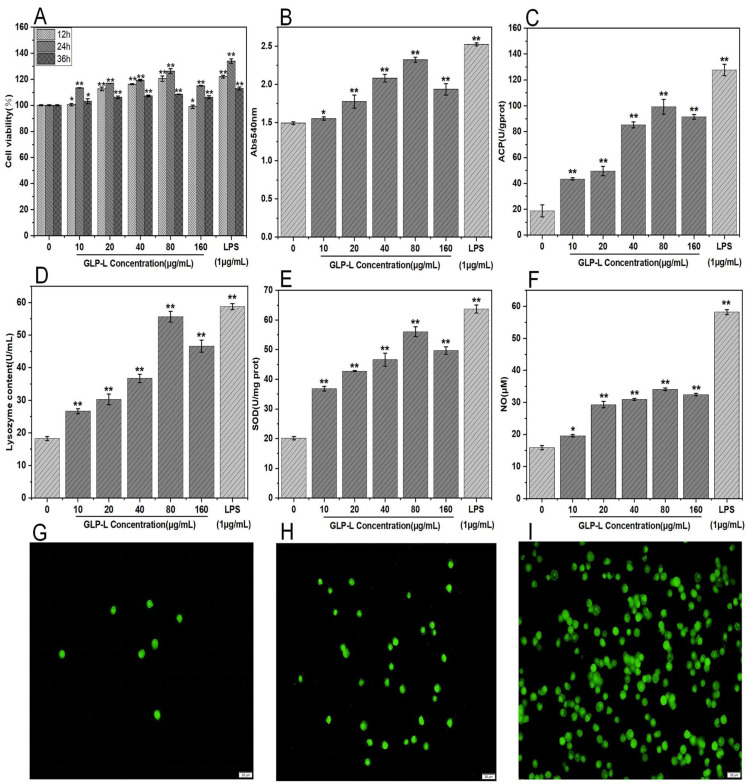
Effects of GLP-L on RAW264.7 cells: cell proliferation (**A**); phagocytosis (**B**); ACP activity (**C**); LZM activity (**D**); SOD activity (**E**); NO production (**F**); AO staining of 0 μg/mL of GLP-L (**G**); AO staining of 40 μg/mL of GLP-L (**H**); AO staining of 80 μg/mL of GLP-L (**I**). Data are presented as mean ± SD (*n* = 3). * *p* < 0.05 and ** *p* < 0.01 versus blank control group. ACP stands for acid phosphatase; LZM stands for lysozyme; SOD stands for superoxide dismutase; NO stands for nitric oxide; AO stands for acridine orange.

The present study utilised the CCK-8 assay to evaluate the effects of varying concentrations of GLP-L on the proliferative activity of RAW264.7 cells. The experiments were conducted at three time intervals (12 h, 24 h, and 36 h) using lipopolysaccharide (LPS) as a positive control. As demonstrated in Figure 5A, cell viability remained within the range of 80–100% at low concentrations (10-40 μg/mL) across all incubation periods. Proliferation activity exhibited a maximum value of 127% at a concentration of 80 μg/mL. A significant decrease was observed at medium to high concentrations (80–160 μg/mL). This phenomenon may be attributed to a mild inhibitory effect resulting from over-stimulation. A thorough investigation into the interaction effect of action time and concentration demonstrated that the optimal incubation time for GLP-L acting on RAW264.7 cells is 24 h. Within the concentration range of 10–160 μg/mL, GLP-L exhibited no significant toxicity towards the cells, suggesting that this concentration window is well-suited for subsequent immunological activity assays.

The process of phagocytosis by macrophages represents a pivotal component in the innate immune response. This constitutes the primary immune barrier against the invasion of pathogenic microorganisms. The neutral red uptake method was utilised to ascertain the endocytic function of RAW264.7 macrophages. The regulatory effect of GLP-L on their phagocytic activity was evaluated. As demonstrated in Figure 5B, the application of GLP-L for a duration of 24 h resulted in a significant enhancement in phagocytic activity in RAW264.7 cells at concentrations ranging from 10 to 160 μg/mL, as compared to the control group (*p* < 0.01). The Abs_540nm_ value was found to reach 2.32 at 80 μg/mL, indicating the maximal activation effect of GLP-L. Phagocytic activity exhibited a slight decline at 160 μg/mL. The results of this study demonstrated that the level of the subject group was still higher than that of the control group. The results obtained demonstrate that GLP-L effectively activates phagocytosis in RAW264.7 macrophages, thereby strengthening immune defence. The concentration that was determined to be optimal for this effect is 80 μg/mL [32].

ACP, LZM, and SOD were utilised to evaluate molecular-level immune cell activation. As a non-specific immune component, ACP is crucial for innate immunity and the maintenance of physiological homeostasis [33]. As demonstrated in Figure 5C, in comparison with the control group, ACP activity was considerably elevated in all polysaccharide-treated groups. The highest level of this parameter was observed at 80 μg/mL (*p* < 0.01). LZM has been shown to degrade insoluble mucopolysaccharides into soluble glycopeptides and to promote bacterial cell wall lysis. The potential of this substance to enhance immunity is attributable to its capacity to modulate pro-inflammatory factors. As illustrated in Figure 5D, there is an enhancement in LZM activity in the concentration range of 10 to 160 μg/mL, with activity increasing by over threefold at 80 μg/mL (*p* < 0.01). As demonstrated in [34], superoxide radicals are converted into oxygen by SOD, thus reducing oxidative stress and associated cellular damage. As demonstrated in Figure 5E, an increase in polysaccharide concentration was observed to result in elevated levels of SOD, reaching a maximum at 80 μg/mL (*p* < 0.01). The stimulation of GLP-L resulted in an increase in SOD activity to 56.05 U/mg protein. This finding suggests that it can effectively enhance the antioxidant capacity of cells.

Nitric oxide (NO) has been identified as a pivotal signalling mediator in immune responses, regulating immune cell activation, pathogen clearance, and inflammatory homeostasis. The Griess method was utilised to quantify nitric oxide (NO) levels. The assay was conducted on culture supernatants collected from RAW264.7 macrophages after treatment with different concentrations of GLP-L for 24 h (Figure 5F). Within the range of 10–80 μg/mL, there was a continuous increase in NO secretion with rising polysaccharide concentration. At a concentration of 80 μg/mL, the level of nitric oxide reached 34.11 μM, which is approximately 2.15 times that of the blank control group (15.89 μM). This finding suggests that the maximal immune activation effect was observed at this dose. The present study demonstrated that an increase in the concentration of GLP-L resulted in a reduction in NO release. This phenomenon may be attributed to the deleterious effects of excessive nitric oxide (NO) on cell function, resulting in a negative feedback loop. The substance under discussion was demonstrated to contribute to the maintenance of immune balance by means of regulating the survival of macrophages. In addition, it was shown to play a role in the prevention of excessive immune activation and tissue damage [35]. In summary, GLP-L effectively activated RAW264.7 macrophages and enhanced immune defence by promoting NO release, with optimal performance at 80 μg/mL. This concentration-dependent effect indicates that, in application, the precise control of the dose is essential to avoid excessive immune damage and to maintain an ideal activation state.

Acridine orange (AO) staining was utilised to evaluate cellular activity [36]. Figure 5G represents the blank control group, Figure 5H corresponds to the 40 μg/mL of GLP-L treatment group, and Figure 5I corresponds to the 80 μg/mL of GLP-L treatment group. As demonstrated in Figure 5G–I, the cells in the blank control group exhibited a small, round, and uneven shape. Following treatment with 80 μg/mL of GLP-L, an increase in cell size and number was observed, accompanied by a notable enhancement in green fluorescence intensity within the nuclei. These observations indicated that RAW264.7 macrophages were activated from a resting state to an active functional state.

TNF-α, IL-1β, IL-6, and iNOS are core functional genes that govern the immune activation of macrophages [37,38,39,40]. Furthermore, they function as pivotal regulators, thereby establishing a connection between cellular immune responses and inflammatory reactions. The levels of mRNA expression exhibited a high degree of correlation with the activation status and response intensity of immune cells. The upregulation of the transcription of these genes is indicative of effective immune cell activation and the initiation of innate immunity. Concurrently, it has been demonstrated to exert a substantial effect on immune function, with particular regard to the defence against and elimination of pathogens [41].

**Figure 6:**
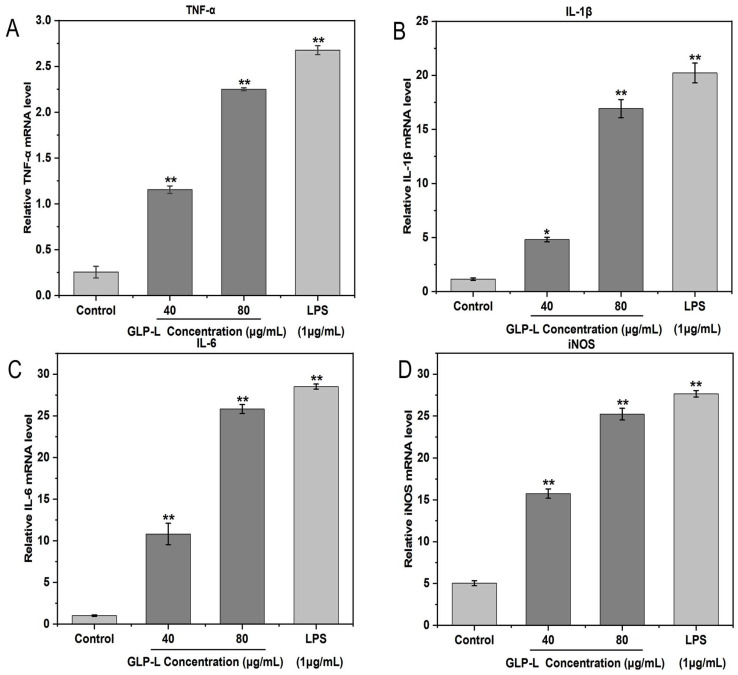
Relative mRNA expression levels were quantified by RT-PCR. TNF-α (**A**); IL-1β (**B**); IL-6 (**C**); iNOS (**D**). Data are means ± SD (*n* = 3). * *p* < 0.05 and ** *p* < 0.01 compared to control group. TNF-α is Tumour Necrosis Factor alpha; IL-1β is Interleukin-1 beta; IL-6 is Interleukin-6; iNOS is Inducible Nitric Oxide Synthase.

As demonstrated in Figure 6, all of the samples that were examined in this study significantly induced the secretion of cytokines in RAW264.7 macrophages. The secretion levels of key pro-inflammatory cytokines in the LPS positive control group and the 80 μg/mL high-concentration GLP-L treatment group were significantly higher than those in the 40 μg/mL low-concentration GLP-L treatment group. The cytokine secretion levels of the three aforementioned groups were found to be significantly higher than those of the blank control group (*p* < 0.01). Among the groups examined, the expression level of the 80 μg/mL GLP-L treatment group was the highest, and this concentration exhibited the strongest immune-stimulating activity. It was demonstrated that GLP-L exhibited a significantly stronger stimulatory effect than the blank control group at each concentration of polysaccharide. The upregulation of immune-related gene expression induced by GLPs is closely related to their chemical structural characteristics. This upregulation is also associated with the specific recognition and binding with pattern recognition receptors of macrophages. GLP-L has been demonstrated to regulate cytokine mRNA expression. This phenomenon may be attributed to the high affinity of D-mannose residues for TLR4 and MR on the surface of RAW264.7 macrophages [42]. It has been demonstrated that the binding of GLP to these receptors results in the activation of downstream signalling pathways, including MyD88/NF-κB and PI3K/Akt. These pathways subsequently facilitate the transfer of transcription factors to the nucleus, thereby initiating the transcription of pro-inflammatory cytokine genes (IL-6 and TNF-α) and the iNOS gene [43,44].

In conclusion, the enzyme extraction process combined with the steam explosion pretreatment of *Ganoderma lucidum* breaks down the dense structure of the raw materials, thereby releasing more crude polysaccharides. The yield of crude polysaccharide is 15.39%, which is 6.96 times that of the traditional water extraction method (2.21%). It was demonstrated that the combined extraction method results in a higher rate of extraction than the water-alone extraction method. The purified homogeneous polysaccharide was designated GLP-L, with a molecular weight of 5.59 × 10^6^ Da. The composition of the substance is D-mannose, D-glucose, D-xylose, and D-galactose, with molar percentages of 52.76%, 36.98%, 6.39%, and 3.87%, respectively. The primary structural chain was identified as follows: 2)-β-D-Gal-(1→4)-α-D-Xyl*p*-(1→3,4)-α-D-Man*p*-(1→6)-β-D-Glc*p*-(1→4)-β-D-Man*p*-(1→4)-α-D-Glc*p*-(1→. The effect of GLP-L on immunomodulatory activity was evaluated through the RAW264.7 macrophage model, with the results showing an enhancement in phagocytic activity, enzyme activities, nitric oxide production, and cytokine secretion. This study provides substantial scientific evidence for enhancing the yield of crude polysaccharides and improving the utilisation of GLPs.

## 3. Material and Methods

*Ganoderma lucidum* species were provided by Jiangsu Alphay Bio-Technology Co., Ltd. (Nantong, China). Cellulase, hemicellulase and pectinase were supplied by Shanghai Yuanye Bio-Technology Co., Ltd. (Shanghai, China). Sephadex G-200 and DEAE-52 were obtained from Beijing Ruida Henghui Technology Co., Ltd. (Beijing, China). The monosaccharide standards were acquired from Sigma-Aldrich (St. Louis, MO, USA). The T-series Dextran (T10, T40, T70, T110, T1000, T2000) and Dulbecco’s Modified Eagle Medium (DMEM) were purchased from Beijing Solarbio Co., Ltd. (Beijing, China). The RAW264.7 murine macrophage cell line was obtained from the Innovation Team for Edible and Medicinal Fungi and Health Products, College of Food Science and Engineering, Tianjin University of Science and Technology (Tianjin, China). Assay kits for acid phosphatase (ACP), lysozyme (LZM), superoxide dismutase (SOD), and lipopolysaccharides (LPSs) were purchased from Nanjing Jiancheng Bioengineering Institute (Nanjing, China). The nitric oxide (NO) assay kit was provided by Beyotime Biotechnology (Haimen, China). All the other chemical reagents and drugs used in this study were of analytical pure grade.

### 3.1. Extraction of Polysaccharides

The *Ganoderma lucidum* powder was subjected to a series of treatments, the first of which was a steam explosion process. This process was carried out using a system designed and manufactured by Zhengdao Bioenergy Co., Ltd. (Hebi, China). The raw material powder was defatted by soaking it in 95% ethanol and then dried at room temperature [45]. The sample was subjected to compound enzymatic hydrolysis with cellulase, hemicellulase and pectinase under optimised conditions. After centrifugation, ethanol precipitation, Sevag deproteinisation and freeze-drying, the crude polysaccharide GLP-CL was obtained. For comparison, the raw material without steam explosion was extracted by conventional hot water to prepare GLP-CW.

### 3.2. Purification of Polysaccharides

GLP-CL was purified successively by DEAE-52 column chromatography and a Sephadex G-200 gel column (Ruidahenghui technology, Beijing, China). The purified fractions were obtained and designated as GLP-L. The purity and molecular weight (Mw) of the polysaccharides in GLP-L were analysed by high-performance liquid chromatography (HPLC). The obtained data were then recorded. A standard curve was constructed [46]. The absence of protein residues was verified by means of an ultraviolet–visible spectrophotometer (Shimadzu-UV3600, Shimadzu, Tokyo, Japan). The total sugar content was determined by the phenol–sulfuric acid method [47]. The uronic acid content was determined using the meta-hydroxybiphenyl method [48,49].

### 3.3. Structural Characterisation of GLP-L

#### 3.3.1. Monosaccharide Composition Analysis

GLP-L was subjected to acid hydrolysis and derivatisation. Gas chromatography–mass spectrometry (GC/MS, Shimadzu, Japan) was adopted for detection [50]. GLP-L (5 mg) was placed in a 10 mL stoppered test tube. A trifluoroacetic acid (TFA) solution (2 mL, 2 mol/L) was then added. The mixture was thoroughly mixed and then subjected to closed hydrolysis at 110 °C in an oil bath for 3 h. This was followed by a series of evaporation steps under nitrogen and subsequent rinsing with methanol, the objective of which was to ensure the complete removal of TFA. A pyridine solution was prepared, comprising inositol hexaacetate and hydroxylamine hydrochloride. The hydrolysed sample and the mixed standard monosaccharide samples were subjected to heating at 90 °C for a duration of 30 min. Subsequently, acetic anhydride was added. The reaction was sustained under identical conditions. The solution was then dried and subsequently redissolved in dichloromethane (DCM). The samples that had been filtered through a 0.22 μm membrane were then analysed by GC/MS.

#### 3.3.2. Fourier Transform Infrared (FT-IR) Analysis

GLP-L (1 mg) was mixed with the KBr powder (150 mg). The mixture was then compressed into a uniform and transparent thin sheet. The spectrum was obtained by means of Fourier transform infrared spectroscopy (FT-IR) (Bruker, Bremen, Germany) within the wavenumber range of 4000–400 cm^−1^ [51].

#### 3.3.3. Methylation Analysis

GLP-L was dissolved in Dimethyl sulfoxide (DMSO). Sodium hydride (NaH) was then added, and the mixture was subjected to ultrasonic treatment in a dark environment. The reactants were then mixed with iodomethane (CH_3_I), and the resultant mixture was subjected to ultrasonic treatment at 20 °C. Following a drying process with N_2_, the methylation process was repeated until completion. The addition of deionised water was followed by the extraction of the sample with DCM. The solvent was then evaporated to yield the methylated product. TFA (2 mL, 2 mol/L) was added to the mixture, which was then heated at 110 °C for 3.5 h. The residue was dissolved in 2 mL of water after drying. Sodium borohydride (NaBH_4_) was then added, and the mixture was left to stand for a period of time. The pH was then adjusted to 5.0 with acetic acid, after which excess reagents were removed. Subsequently, a quantity of 2 mL of methanolic solution was added, along with a drop of acetic acid. The sample was subjected to a process involving the mixing of acetic anhydride, followed by treatment at 110 °C for a duration of 1 h. Subsequent to the drying process using nitrogen, the addition of DCM to the sample was undertaken. The substance was then subjected to GC/MS analysis.

#### 3.3.4. Nuclear Magnetic Resonance (NMR) Analysis

GLP-L (60 mg) was dissolved in D_2_O (1.2 mL). The measurement was conducted under controlled environmental conditions. An NMR spectrometer (Thermo Nicolet, Madison, WI, USA) was used to record ^1^H, ^13^C, HMBC, COSY and HSQC spectra.

### 3.4. Immunomodulatory Activity

#### 3.4.1. Cultivation of Cells

RAW 264.7 cells were cultivated in a high-glucose DMEM, which was enriched with 10% foetal bovine serum. The cells were maintained at a temperature of 37 °C in an atmosphere containing 5% CO_2_.

#### 3.4.2. Cell Viability

The assessment of cell viability was conducted by means of the Cell Counting Kit-8 (CCK-8). The cells were treated with GLP-L at concentrations ranging from 0 to 160 μg/mL. Lipopolysaccharide (LPS) at 1 μg/mL was administered in the same manner as a positive control. Absorbance was measured at a wavelength of 450 nm using the provided kit following 12, 24, and 36 h of treatment. Viability was then normalised to the blank control and expressed as a percentage [52]. Cell viability was analysed using Equation (1).

(1) $$\begin{array}{c} Cell\;viability(\%)=\frac{\left(\mathrm{As}-\mathrm{Ab}\right)}{\left(\mathrm{Ac}-\mathrm{Ab}\right)}\times 100\% \end{array}$$

where *As* is the absorbance value read for the experimental well; *Ac* is the value from the blank control well; *Ab* is the value of the blank well.

#### 3.4.3. Determination of Immunomodulatory Activity Parameters

##### Cell Pretreatment

RAW 264.7 cells were seeded into 96-well plates and then left to incubate for a period of 24 h. Following this, cell morphology and the extent of cell fusion were evaluated by means of an inverted microscope. The medium was replaced. The establishment of a blank control group (fresh culture medium) and a positive control group (1 μg/mL LPS) was undertaken. The experimental groups were treated with GLP-L at concentrations ranging from 10 to 160 μg/mL. The following specified parameters were evaluated after 24 h of cultivation.

##### Determination of Phagocytic Capacity

After aspirating the medium, a neutral red solution was applied for a period of 4 h in a dark environment. The preceding solution was substituted with cell lysis buffer. Absorbance was measured at a wavelength of 540 nm [53]. The effects of GLP-L on the phagocytic function of the cells were evaluated.

##### Determination of ACP, LZM and SOD

The pretreated cells were then rinsed with phosphate-buffered saline (PBS). The cells were lysed using cell lysis buffer containing PMSF. The superior portion of the sample was collected subsequent to centrifugation. The protein content was determined. The activities of acid phosphatase (ACP), lysozyme (LZM), and superoxide dismutase (SOD) were determined by using commercial assay kits. The values were recorded and subsequently calculated.

##### Cell Morphology Observation

After the completion of the culture, the cells were subjected to staining with acridine orange (AO) in hydrochloric acid. The nuclear morphology of these cells was observed under a fluorescence microscope.

##### Assessment of Nitric Oxide (NO) Release

Following the incubation period, the levels of nitric oxide (NO) in the serum were determined by means of the Griess reagent assay [54].

##### RT-qPCR Assay

RT-qPCR was utilised to assess the expression levels of pro-inflammatory cytokine genes TNF-α, IL-1β, IL-6, and iNOS. Total RNA was isolated using TRIzol reagent (Life-iLab, Shanghai, China) under aseptic and enzyme-free conditions. Subsequently, cDNA was synthesised using the mix (Accurate Biotechnology, Changsha, China) and gene-specific primers. The corresponding reaction mixture (purchased from Accurate Biotechnology, Changsha, China) and the primers listed in Supplementary Table S1 were added. qPCR detection was then performed using an RT-qPCR system (Bio-Rad Laboratories, Shanghai, China). Subsequent to this, the normalisation of all gene expression levels was conducted in relation to the Ct value of the internal reference gene, Gapdh. The 2^−ΔΔCt^ method was the chosen approach for the analysis of the data [55].

## 4. Statistical Analysis

The mean and standard deviation were calculated and analysed based on three independent parallel experiments. Statistical analysis was conducted utilising SPSS 26.0 software. In order to facilitate intergroup comparisons, a one-way ANOVA was employed, with the subsequent implementation of Tukey’s multiple comparison test. The error bars in all figures represent the standard deviation (SD, *n* = 3). Significance marks were employed in a uniform manner, and it is evident that the *p*-value is less than 0.05 and less than 0.01.

## Figures and Tables

**Table 1:** Methylation analysis of GLP-L.

Linkage Pattern	Methylated Sugar Residues	Mass Fragments (*m*/*z*)	Molar Percentages (%)
T-Man(*p*)	1,5-Di-O-acetyl-1-deuterio-2,3,4,6-tetra-O-methyl-D-mannitol	43,71,87,99,118,129,161, 162,205	3.16
2-Gal(*f*)	1,4-Di-O-acetyl-1-deuterio-2,3,5,6-tetra-O-methyl-D-galactitol	43,71,87,101,117,129,161,191,233	4.69
4-Glc(*p*)	1,4,5-Tri-O-acetyl-1-deuterio-2,3,6-tri-O-methyl-D-glucitol	43,71,85,102,118,129,160,207,233	8.62
4-Man(*p*)	1,4,5-Tri-O-acetyl-1-deuterio-2,3,6-tri-O-methyl-D-mannitol	43,71,87,118,129,173,203,233	42.45
6-Glc(*p*)	1,5,6-Tri-O-acetyl-1-deuterio-2,3,4-tri-O-methyl-D-glucitol	43,71,87,118,129,160,189,207,233	30.70
4-Xyl(*p*)	1,4,5-Tri-O-acetyl-1-deuterio-2,3-di-O-methyl-D-xylitol	43,71,87,118,129,160,189	6.11
3,4-Man(*p*)	1,3,4,5-Tetra-O-acetyl-1-deuterio-2,6-di-O-methyl-D-mannitol	43,71,87,118,127,201,305	4.26

**Table 2:** Chemical shifts for the residues of GLP-L.

Code	Glycosyl Residues	Chemical Shifts (ppm)					
		H1/C1	H2/C2	H3/C3	H4/C4	H5/C5	H6/C6
A	β-D-Man*p*-(1→	4.64	3.4	3.67	3.54	3.98	3.79
		102.72	74.14	73.22	66.93	76.76	60.80
B	→2)-β-D-Gal*f*-(1→	5.06	4.00	4.14	3.91	3.78	3.63
		106.27	83.20	75.47	82.41	72.74	62.87
C	→4)-α-D-Glc*p*-(1→	4.98	3.32	3.91	3.56	3.22	3.56
		98.21	73.54	74.99	78.86	73.6	62.74
D	→4)-β-D-Man*p*-(1→	4.78	3.90	3.55	3.91	3.37	3.59
		100.30	71.17	74.51	82.41	76.08	62.8
E	→6)-β-D-Glc*p*-(1→	4.42	3.19	3.86	3.72	3.56	4.10
		102.72	75.64	78.54	70.34	78.86	69.76
F	→4)-α-D-Xyl-(1→	4.87	3.93	3.88	4.05	-	-
		97.89	76.19	66.45	69.67	-	-
G	→3,4)-α-D-Man*p*-(1→	4.92	3.96	3.92	3.64	3.86	3.86
		102.24	69.97	72.41	75.64	73.13	60.67
H	α reducing Glc*p*	5.12	3.45	3.56	3.35	3.79	3.46
		92.08	70.04	71.36	69.51	74.51	62.42
I	β reducing Glc*p*	4.54	3.2	3.49	3.29	3.64	3.46
		95.79	73.94	72.03	69.51	76.02	62.45

## Data Availability

The original contributions presented in this study are included in the article/Supplementary Materials. Further inquiries can be directed to the corresponding authors.

## Supplementary Materials

The following supporting information can be downloaded at: https://www.mdpi.com/article/10.3390/molecules31111864/s1. Figure S1. The molecular weight distribution of GLP-CL. Figure S2. Elution profile of DEAE-52. Figure S3. Molecular weight distribution after DEAE-52. Figure S4. Elution profile of Sephadex G-200. Figure S5. UV spectrum of GLP-L. Figure S6. The standard curve of molecular weight. Figure S7. Glucose standard curve. Figure S8. Galacturonic acid standard curve. Figure S9. (A) Total ion profile of partially methylated alditol acetates of GLP-L obtained by GC-MS, (B) MS fragments and deduced residues. Table S1. Primer sequence of GLP-L.
