# Supplementary material for: Enzyme-Assisted Extraction of Polysaccharides from Steam-Exploded Ganoderma lucidum and Its Yield, Structural Characterisation, and Immunomodulatory Activity
Source: Molecules. 2026 May 29;31(11):1864. doi: 10.3390/molecules31111864 (PMC13257831; doi:10.3390/molecules31111864)
Supplement: Supplementary file 1 [file molecules-31-01864-s001.zip › molecules-4304332-supplementary.pdf]

Supporting Information

1.Methods

1.1 Extraction of polysaccharides

The *Ganoderma lucidum* powder was subjected to a series of treatments, the first of which was a steam explosion process. This process was carried out using a system designed and manufactured by Zhengdao Bioenergy Co. Ltd. (Hebi, China). The explosion was initiated at a pressure of 1.5 MPa and lasted for 150 s. The raw material powder was defatted by soaking in 95% ethanol and then dried at room temperature. The raw material (1 g) was mixed with 30.00 mL of phosphate buffer solution (pH 5.35). Subsequently, the addition of compound enzymes, comprising cellulase, hemicellulase and pectinase, occurred in a sequential manner, at enzyme-substrate ratios of 1800 U/g, 1800 U/g and 800 U/g, correspondingly. The reaction mixture was stirred at a constant temperature of 55°C for 2 h, and then transferred to a water bath at 90°C and heated for 10 min to terminate the enzymatic reaction. The upper layer was collected by means of centrifugation, after which anhydrous ethanol was added at a volume ratio of 4:1 (v/v). The extract was left to stand at 4°C for 24 h. Thereafter, the precipitate was separated and redissolved. The protein was removed by the Sevag reagent method. Subsequent to vacuum freeze-drying, the crude polysaccharide was obtained and designated GLP-CL. In order to elucidate the effect of this method on the yield, the raw material without steam explosion pretreatment was subjected to water extraction (80°C, 2 h, 1:20 g/mL). The crude polysaccharide obtained was designated as GLP-CW, and its yield was compared with that of GLP-CL.

1.2. Purification of polysaccharides

The purification of GLP-CL was achieved through DEAE-52 column chromatography (column size: 2.6 cm × 40 cm, flow rate: 1.5 mL/min). The elution system comprises water and NaCl solutions, each with a concentration gradient of 0.1, 0.2, or 0.3 mol/L. For each elution step, 20 fractions (6 mL per fraction) were collected. The components were then subjected to a further purification process via a Sephadex G-200 gel column (2.6 cm × 40 cm). The mobile phase was water, with a flow rate of 0.30 mL/min. A total of 40 eluate fractions (with a volume of 1.8 mL per fraction) were collected during this process. Fractions corresponding to the main peaks of the elution curve were harvested. The purified fraction was obtained and designated as GLP-L.

The homogeneity of the polysaccharides of GLP-L and the molecular weights (Mw) of GLP-L and standard dextran (T-10, T-40, T-70, T-500, T-2000) were analysed by high-performance liquid chromatography (HPLC). The analysis was conducted utilising a TSK Gel G4000PWXL column (7.8 mm × 300 mm) in conjunction with a differential refractive index detector (G7162A). The parameters were set to 35°C for the column and 30°C for the detector. The retention time was recorded at a flow rate of 0.6 mL/min. The standard curve was constructed. The absence of protein residues was verified using a UV-Vis spectrophotometer (Shimadzu-UV3600, Japan). The total sugar content was determined by means of the phenol-sulfuric acid method. The uronic acid content was determined using the meta-hydroxybiphenyl method.

2.Figure.s

Figure. Captions

Figure. S1. The molecular weight distribution of GLP-CL.

Figure. S2. Elution profile of DEAE-52

Figure. S3. Molecular weight distribution after DEAE-52

Figure. S4. Elution profile of Sephadex G-200

Figure. S5. UV spectrum of GLP-L

Figure. S6. The standard curve of molecular weight.

Figure. S7. Glucose standard curve.

Figure. S8. Galacturonic acid standard curve.

Figure. S9. (A) Total ion profile of partially methylated alditol acetates of GLP-L obtained by GC-MS, **(B)** MS fragments and deduced residues.

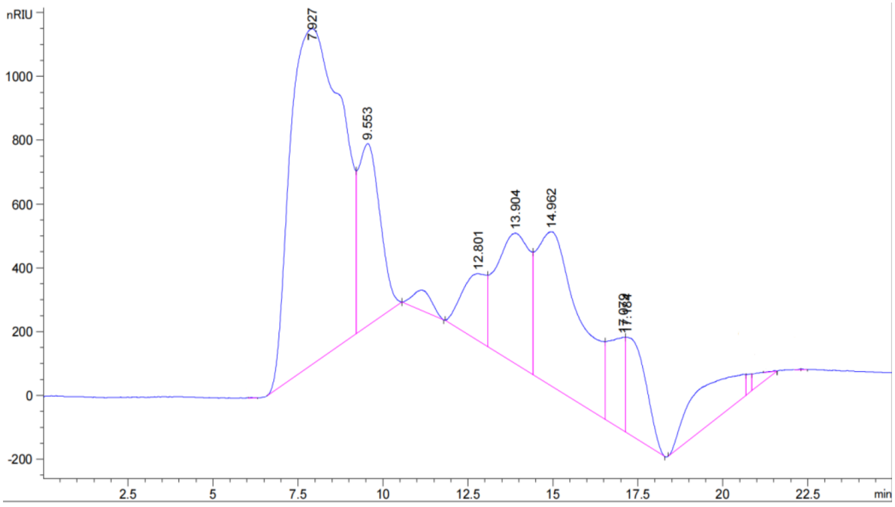

Figure. S1. The molecular weight distribution of GLP-CL

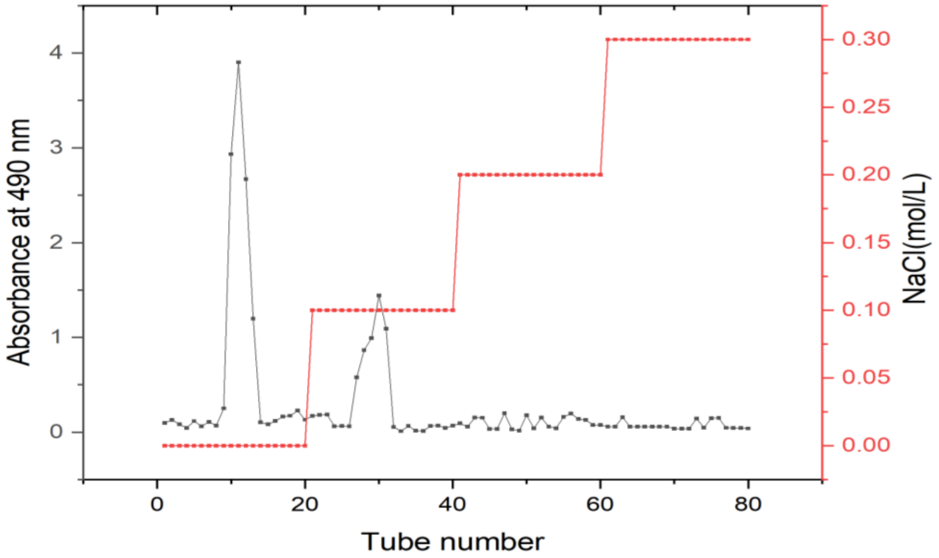

Figure. S2. Elution profile of DEAE-52

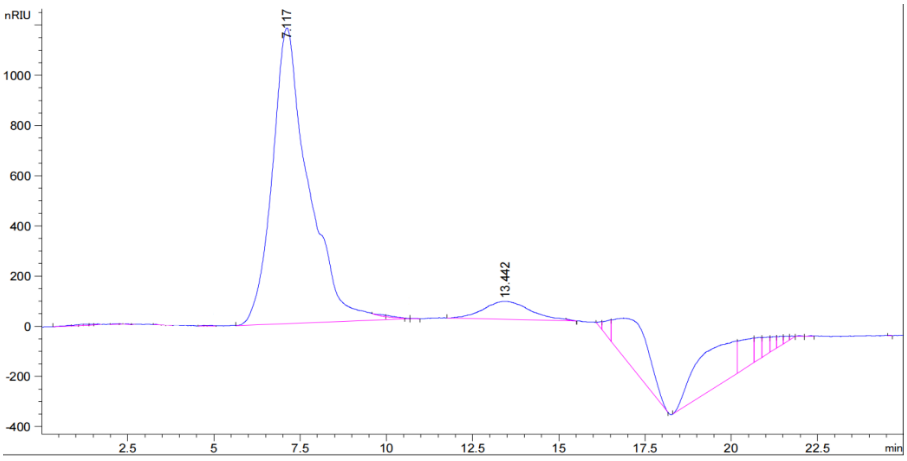

Figure. S3. Molecular weight distribution after DEAE-52

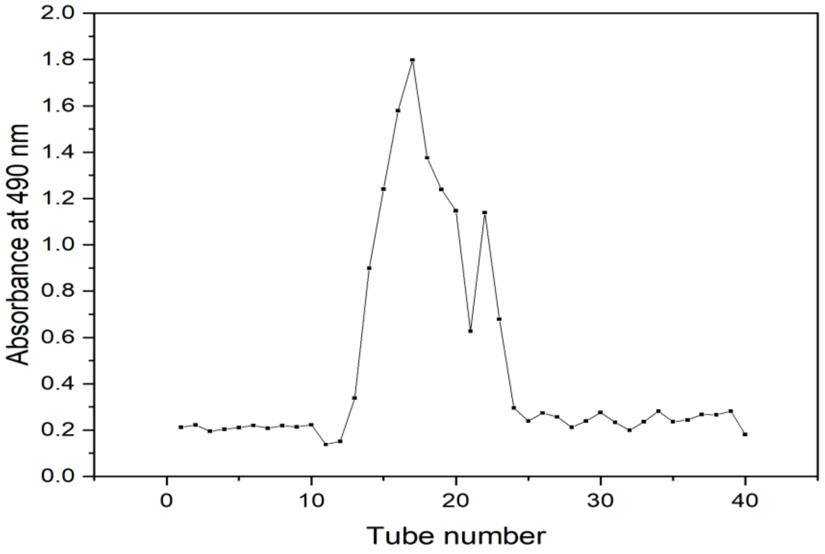

Figure. S4. Elution profile of Sephadex G-200

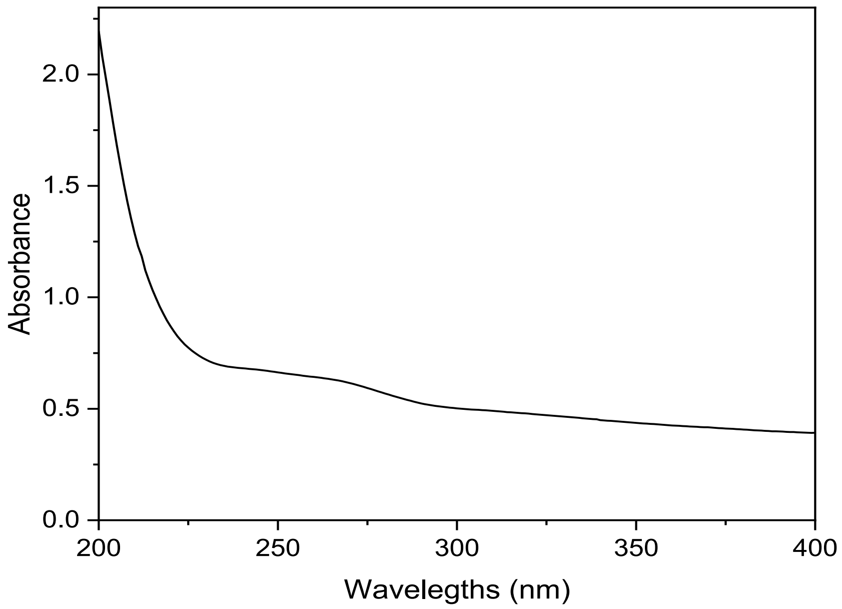

Figure. S5. UV spectrum of GLP-L

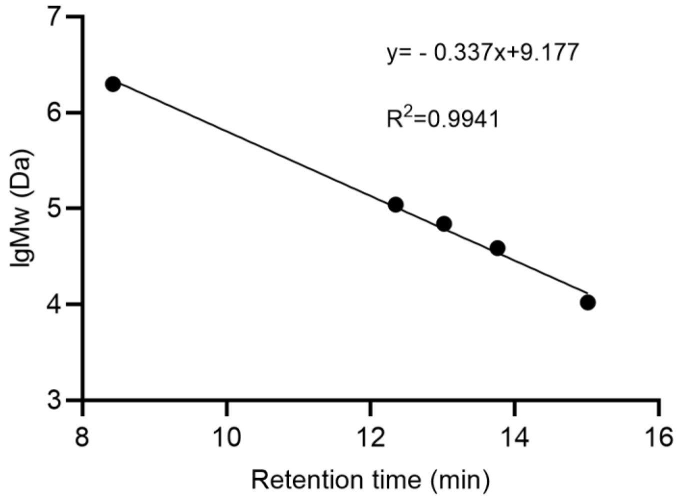

Figure. S6. The standard curve of molecular weight.

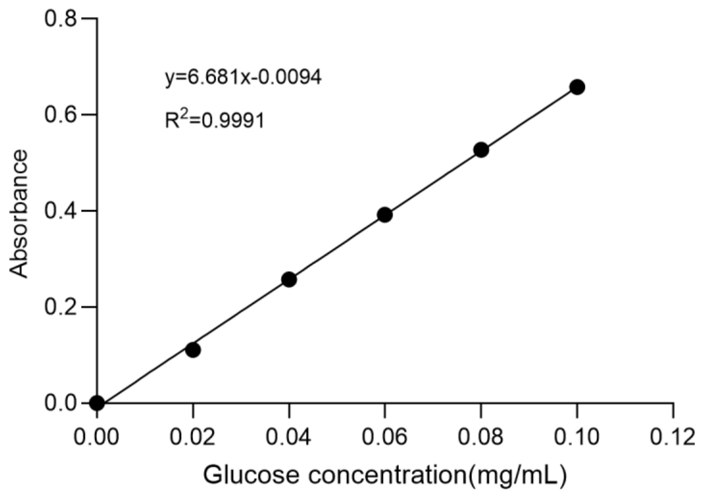

Figure. S7. Glucose standard curve.

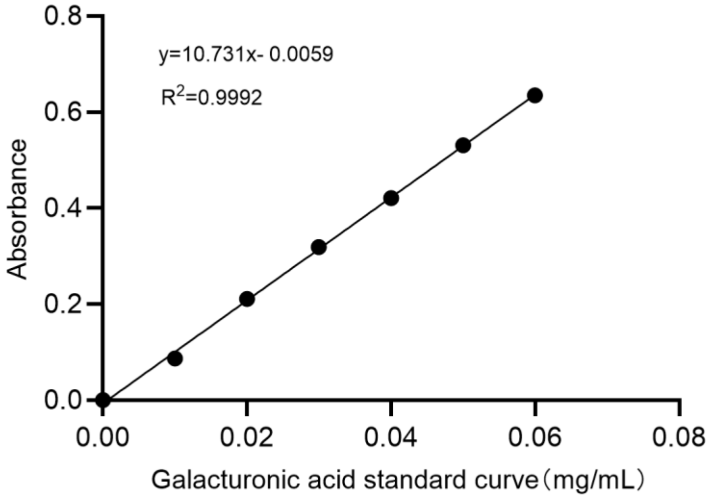

Figure. S8. Galacturonic acid standard curve.

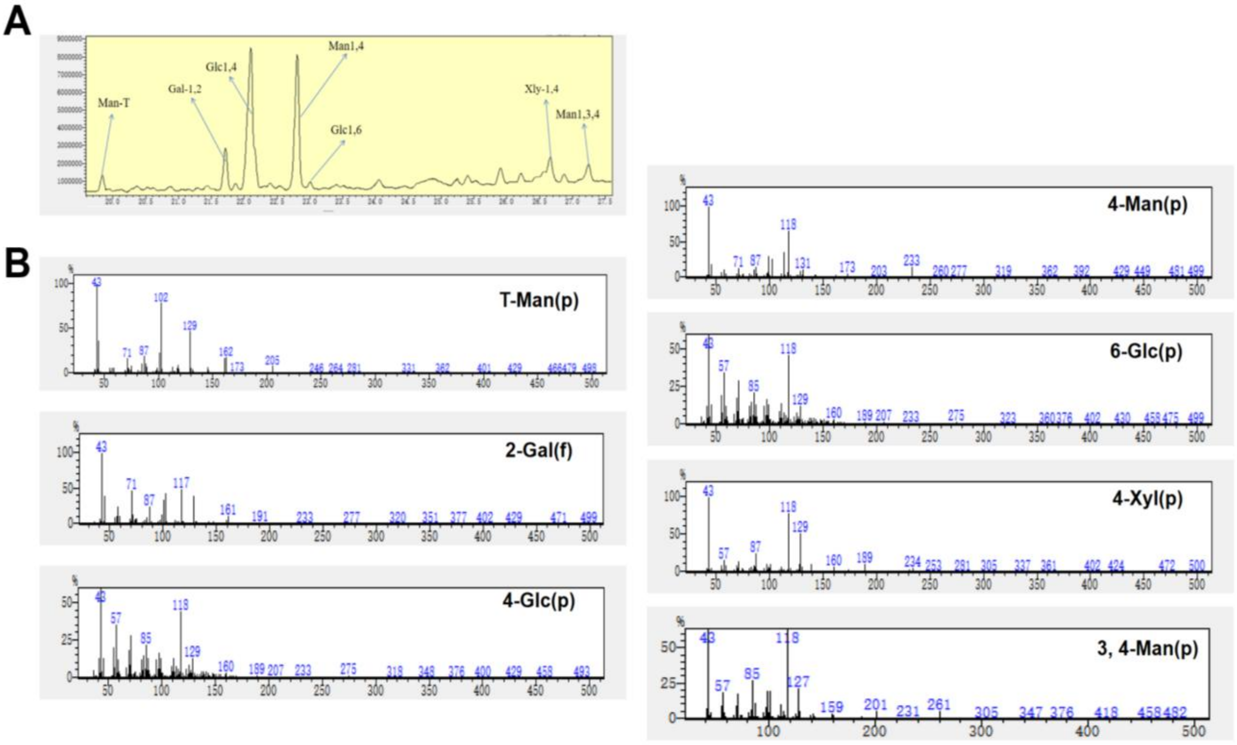

Figure. S9. (A) Total ion profile of partially methylated alditol acetates of GLP-L obtained by GC-MS, **(B)** MS fragments and deduced residues.

3.Tables

Table S1 Primer sequence of GLP-L.

Gene	Forward Primer Sequence(5’ - 3’)	Reverse Primer Sequence(5’ - 3’)
Gapdh	ACCCTTAAGAGGGATGCTGC	CCCAATACGGCCAAATCCGT
INOS	TCTAGTGAAGCAAAGCCCAAC	CACATACTGTGGACGGGTCG
TNF-α	GATCGGTCCCCAAAGGGATG	CCACTTGGTGGTTTGTGAGTG
IL-1β	TGCCACCTTTTGACAGTGATG	TGATGTGCTGCTGCGAGATT
IL-6	TGGTCTTCTGGAGTACCATAGC	TGTGACTCCAGCTTATCTCTTGG
